# Ultrasound‐guided Erector Spinae Plane Block (US‐ESPB)—Anesthetic block: Case report

**DOI:** 10.1002/ccr3.3253

**Published:** 2020-09-10

**Authors:** Antonio Coviello, Maria Vargas, Gaetano Castellano, Alfredo Maresca, Giuseppe Servillo

**Affiliations:** ^1^ Department of Anesthesiology and Intensive Care Medicine Policlinico Federico II University Hospital Naples Italy; ^2^ Department of Anesthesiology and Intensive Care Medicine Policlinico‐Federico II University Hospital Naples Italy; ^3^ Department of Anesthesiology, Intensive Care and Pain Medicine Gemelli Molise Hospital of the Catholic University of the Sacred Heart Campobasso Italy

**Keywords:** ESP ‐ Erector Spinae Plane, ESP anesthesia, thoracic wall surgery, US‐ESPB ‐ erector spinae block

## Abstract

The Ultrasound‐guided Erector Spinae Plane Block (US‐ESPB), used as an anesthesiological block, could represent a safe and effective alternative for thoracic wall surgery especially in fragile, obese patients and those with respiratory and/or hemodynamic problems.

## Graphical abstract

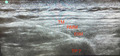

## 1 INTRODUCTION

Ultrasound‐guided Erector Spinae Plane Block, at T5 transverse process, was recently described as a technique for providing thoracic analgesia.^1^, ^2^, ^3^

From recent literature, it is known that the injection of local anesthetic (LA) into the deep fascial plane to erector spinae muscle (the erector spinae plane, ESP) at the level of the T5 transverse process can produce profound analgesia of the ipsilateral hemithorax.^4^, ^5^

Anatomical dissection indicates that the likely action mechanism is the diffusion of LA anteriorly through the connective tissues and ligaments spanning the adjacent transverse processes and into the vicinity of the spinal nerve roots (consistent with other reports of successful analgesia following injection into a similar tissue plane in the thorax^6^, ^7^).

According to the literature, the technique that allows the best spread of LA consists in the injection of LA in the deep interfascial plane of the spinae erector muscle; in this way, it spreads out widely between the erector muscles of the spine and the costotransverse process in the paravertebral space, between the intervertebral foramina, in the epidural space, and near the ipsilateral sympathetic chain.^8^

The visceral and somatic analgesic effects provided by the ESPB likely result from both transforaminal and epidural spread. This explains the visceral pathway and the multiple spinal segmental blockade (occasionally bilateral) through the circumferential epidural spread. Although we only observed superficial intercostal muscle spread over several spinal levels, subsequent deep penetration to reach spinal nerves cannot be ruled out.^9^

One of the advantages of the ESP block is that it gains indirect access to the paravertebral space and provides analgesia without the potential needle‐pleura interaction and the consequent risk of pneumothorax. The technique is performed identifying the erector spinae muscle above transverse process, directing the needle toward the bone, and injecting LA. Another advantage of the ESP block is that it could also be performed, quite simply, in the obese patient, making it an attractive option. Moreover, this technique can be performed by all, quickly and simply, as it is reliable and consistent, allows opioids sparing, and has minimal complications. ^10^, ^11^

Erector spinae plane is widely used, particularly in thoracic surgery, for intra‐ and postoperative analgesia. Erector Spinae Plane block that determines anesthesia and not only analgesia is not diffused in current clinical practice.^12^, ^13^, ^14^ This case report shows that in certain cases, ESP can also be used as an effective and safe anesthesiological alternative.

## 2 CASE REPORT

A 25‐year‐old female patient came to our observation, without comorbidity, to undergo to the exeresis of a capsulated lipoma of about 10 cm of diameter localized under the lower corner of the scapula, under the fascial plane, in the left hemithorax. The patient reported history of awareness during a previous surgery and refused general anesthesia.

The clinical case was presented to the entire surgical/anesthesiological team involved. It was clear that the intervention could not be carried out with local anesthesia practiced by the surgeon. The most appropriate choice seemed to be, initially, performing a paravertebral block (PVB), since it is the only peripheral block described as able to determine an adequate anesthesiological plane to the type of the intervention. Obviously, it is a procedure not free from complications, such as pneumothorax or accidental spinal injection, even if practiced by expert personnel. The ESP block, another peripheral block of the thoracic wall, is described only as an antalgic block. Following a discussion about the potential risks and benefits, the team came to the conclusion that ESP block could be suitable for the planned surgery as an indirect PVB.

Once the extension of the anesthesiological plane, necessary for the type of surgery, was defined with surgical team, and once anesthetic technique was carefully assessed, the level of the puncture, the anesthetic mixture, and the entire anesthesiological conduct were planned.

**FIGURE 1:**
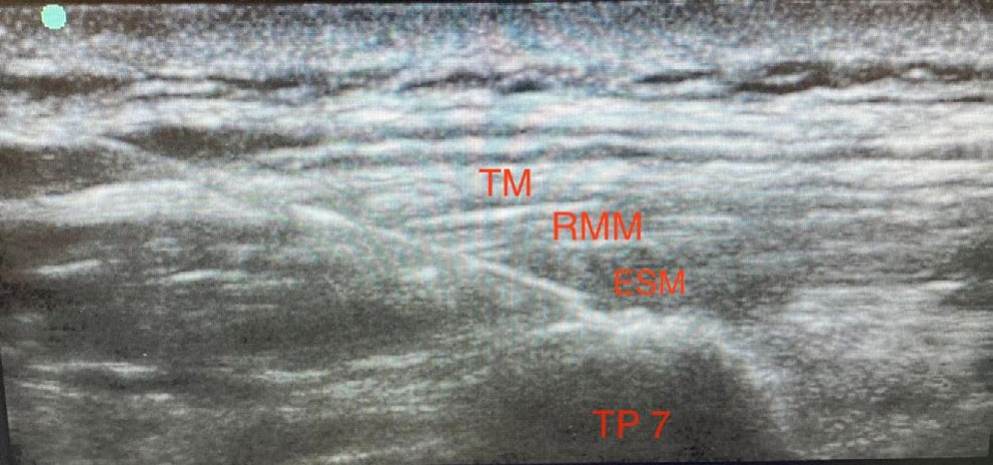
Image from the procedure: a 22‐gauge 50‐mm block needle was inserted in plane, with a cephalad‐to‐caudad direction. ESM, erector spinae muscle; RMM, rhomboid major muscle; TM, trapezius muscle; TP, transverse process

In operating room, the patient was placed in sitting position, a high‐frequency linear probe 12 MHz (Sonosite HLF38x 13‐6 MHz; Fujifilm Sonosite Europe), and a 22 Gauge 50‐mm (Vygon Locoplex) block needle was used. Prescanning transverse and longitudinal views were performed sonographically to identify bone structures, including ribs, spinous processes, laminae, transverse processes, and muscles, including the trapezius, rhomboid, and erector spinae muscles, at the level of the seventh thoracic vertebrae (T7). The probe was positioned longitudinally on the T7 transverse process, approximately 3 cm away from the midline, and anatomical structures were imaged in the sagittal plane. For the ESP block, the same in‐plane technique with ultrasound guidance was used to advance the needle toward the tip of the T7 transverse process, in a cephalic to caudal direction (Figure 1), until contact was made with the transverse process. The correct location of the needle tip in the fascial plane deep to the erector spinae muscles was confirmed by injecting 3 mL of normal saline and observing that the injected fluid lifted the erector spinae muscle off the transverse process without intramuscular injection. After confirming the correct localization of the needle tip, 20 mL of 0.75% ropivacaine and dexamethasone 8 mg was injected.

After 20 minutes from the procedure, a sensory block (Hollmen 4), tested with Pin Prick and Ice tests, was obtained with an extension from T5 to L1 for the entire duration of the surgery (30’) during which the patient was in prone position, in spontaneous breathing, sedated with 0.7 mcg/kg/h of dexmedetomidine 10 minutes before the surgery. The vital parameters were stable throughout the duration of the intervention. The patient was a RASS score −2 throughout the intervention.

In the postoperative, at T1 (2 hour postsurgery), the patient reported Hollmen 3 analgesia; at T2 (6 hour postsurgery) Hollmen 2 analgesia; at T3 (12 hour postsurgery) Hollmen 1 analgesia.

At 24 hour postsurgery, the patient reported a Numeric Rating Scale (NRS) <2, with no analgesic necessity or recourse to rescue dose.

Our anesthesiological choice did not determine any limitation of movement both intra‐ and postsurgery. There were neither nausea, vomiting, nor shivers in the postoperative time. The patient was contactable and cooperative for the entire surgical procedure and for the entire postoperative period. Even the surgeons were satisfied in proceeding with a constant plane of anesthesia that did not alter the anatomical planes.

The patient gave consent to the publication of this case report, intended purely for scientific and clinical purposes, maintaining the privacy of the processing of her personal data.

## 3 DISCUSSION

Our clinical case showed the efficacy and the safety of a multidermatomic anesthesiology plane with the use of LA at anesthetic concentrations, unlike Forero et al that showed a large multidermatomic sensory block with the use of minor LA concentrations. As suggested by Forero in his previous reports, it is due to the diffusion of the anesthetic solution both in the paravertebral space and in the peridural space.^1^, ^2^

Moreover, ESP block provided a long‐lasting analgesia, probably due to the spread of anesthetic solution close to the intervertebral foramina, next to the origin of the dorsal and ventral branches of the thoracic spinal nerves, as suggested by cadaveric investigation by Vidal et al, or by Schwartzmann et al from a magnetic resonance imaging study.^8^, ^9^

Further researches, as also suggested by Fusco et al, should be necessary to confirm whether the ESP block could be, in some cases, an effective alternative to PVB.

Paravertebral block is currently the gold standard for management of chronic thoracic pain. Unfortunately, contraindications such as coagulopathies or anticoagulant therapies and the difficulty in performing the block may determine the exclusion of many patients from this treatment.

Furthermore, the PVB, currently described in literature as the only peripheral block of the posterior thoracic wall able to determine an anesthesiological plane, requires advanced skills and competences for the professional and is burdened with a high risk of complications such as pneumothorax or spinal injection.^6^, ^7^

Esp block is, instead, a basic ecoguidated block that, as described by El‐Boghdadly and al., is simple and safe, having the transverse process as main target.^10^

Chin et al, in their work, performed a ESP block at T7 level in afresh cadaver and assessed the extent of injectate spread using computerized tomography. There was radiographic evidence of spread extending cranially to the upper thoracic levels and caudally as far as the L2‐L3 transverse processes. This metameric extension was considered by us the most suitable to our surgical intervention, so we decided the level of execution of the procedure was at T7.

Our metameric extension, tested with Pin Prick and Ice test before surgery, is comparable to that one shown by Chin et al^4^

Erector spinae plane block compared to a LA performed by surgeon does not alter the surgical planes and improves the outcome of the patient both in terms of analgesia and surgical times.

Considering the side effects that are present following general anesthesia, locoregional anesthesia with analgosedation or for anesthesiological alternatives that could be considered for our intervention, our anesthesiologist choice has not presented any event of nausea, vomiting, shivers, or itch, giving optimal comfort to the patient. Our anesthesiological management has not required the administration of opioids, avoiding the side effects associated with them.

Therefore, the proposed anesthesiological approach may be particularly suitable for fragile patients or for those who may get more benefits, given their comorbities, from an opioid‐free anesthesia. Moreover, it allows to carry out the intervention in the day surgery regime reducing the hospital length of stay and the costs related to it. Therefore, we, like Milone M. et al, for the ultrasound‐guided transversus abdominis plane block, believe that ESP block could be an anesthesia method for wall surgery.^14^

The clinical benefits of the ESP block need to be evaluated on a larger population to further ensure the clinical validity demonstrated in this case report.

In fact, this anesthetic technique could be a valid anesthesiological alternative to be taken into consideration in certain clinical cases, particularly in patients with respiratory and hemodynamic problems.

Further researches are necessary to confirm whether the ESP block could be, in some cases, an effective alternative to PVB.

This is only a case report and there are limitations inherent to this type of publication, such as the danger of overinterpretation. We think this procedure can be generalizable and applicable to similar interventions. However, there are merits of this type of publication such as the detection of novelties and generation of hypotheses.

## 4 CONCLUSION

The ESP block, which is mainly used as an antalgic rather than anesthesiology block, represents a valid anesthetic alternative for thoracic wall surgery without clinically disrupting the respiratory mechanism and without determining any hemodynamic impact.

In our opinion, the direction of the needle is fundamental to address the spread of the anesthetic mixture. In our clinical case, the choice of the cranial caudal approach was well considered before performing the procedure. The position of the patient is quite important for the execution of the block. According to our experience, the sitting position is the best in terms of simplification of the technique.

## CONFLICT OF INTEREST

None declared.

## AUTHOR CONTRIBUTION

All persons who meet authorship criteria are listed as authors, and all authors certify that they have participated sufficiently in the work to take public responsibility for the content, including participation in the concept, design, analysis, writing, or revision of the manuscript. Furthermore, each author certifies that this material or similar material has not been and will not be submitted to or published in any other publication before its appearance in the *Clinical Case Reports*.

Category 1: AC and MV: involved in conception and designed the study. GC and AM: involved in acquisition of data. AC, MV, and GS: analyzed and/or interpreted the data. Category 2: AC, GC, and AM: drafted the manuscript. AC, MV, and GS: critically revised the manuscript for important intellectual content. Category 3: AC, MV, GC, AM, and GS: approved the version of the manuscript to be published.
